# Supplementary material for: Mucilage produced by aerial roots hosts diazotrophs that provide nitrogen in Sorghum bicolor
Source: PLoS Biol. 2025 Mar 3;23(3):e3003037. doi: 10.1371/journal.pbio.3003037 (PMC12136154; doi:10.1371/journal.pbio.3003037)
Supplement: S1 Table — (DOCX) [file pbio.3003037.s007.docx]

**S1 Table.** Sorghum accessions to evaluate aerial root formation in greenhouse and field experiments

| **Accession** | **Collection** | **Origin** |
| --- | --- | --- |
| IS10757 | Minicore | Chad |
| IS11026 | Minicore | Ethiopia |
| IS15170 | Minicore | Cameroon |
| IS15466 | Minicore | Cameroon |
| IS15478 | Minicore | Cameroon |
| IS17348 | ICRISAT | Ethiopia |
| IS23992 | Minicore | Yemen |
| IS24453 | Minicore | South Africa |
| IS25089 | Minicore | Ghana |
| IS28023 | ICRISAT | Yemen |
| IS28025 | ICRISAT | Yemen |
| IS28033 | ICRISAT | Yemen |
| IS28262 | ICRISAT | Yemen |
| IS29078 | ICRISAT | Yemen |
