# Supplementary material for: Mucilage produced by aerial roots hosts diazotrophs that provide nitrogen in Sorghum bicolor
Source: PLoS Biol. 2025 Mar 3;23(3):e3003037. doi: 10.1371/journal.pbio.3003037 (PMC12136154; doi:10.1371/journal.pbio.3003037)
Supplement: S2 Table — Monosaccharide composition was determined by GC–MS. * data obtained from 1.64 mg sample; ** data obtained from a 2.16 mg sample; nd, not detected. (DOCX) [file pbio.3003037.s008.docx]

**S2 Table.** Mucilage sugar composition of accession IS11026 in two independent experiments. Monosaccharide composition was determined by GC-MS. * data obtained from 1.64 mg sample; ** data obtained from a 2.16 mg sample; nd = not detected.

|  | **Replicate 1*** | | **Replicate 2**** | |
| --- | --- | --- | --- | --- |
| **Monosaccharide** | **Amount (μg)** | **% by mole** | **Amount (μg)** | **% by mole** |
| Arabinose | 163.9 | 19.77 | 254.4 | 20.66 |
| Rhamnose | nd | - | nd | - |
| Ribose | nd | - | nd | - |
| Fucose | 124.9 | 13.77 | 224.6 | 16.68 |
| Xylose | 21.1 | 2.54 | 34.1 | 2.77 |
| Glucuronic acid | 75.9 | 7.08 | 121.8 | 7.65 |
| Galacturonic acid | nd | - | nd | - |
| Mannose | 67.8 | 6.81 | 87.1 | 5.89 |
| Galactose | 484.9 | 48.73 | 674.0 | 45.59 |
| Glucose | 12.9 | 1.29 | 11.4 | 0.77 |
| *N*-Acetyl mannosamine | nd | - | nd | - |
| *N*-Acetyl galactosamine | nd | - | nd | - |
| *N*-Acetyl glucosamine | nd | - | nd | - |
| **Total** | 951.4 | 100 | 1,407.4 | 100 |
