# Supplementary material for: Mucilage produced by aerial roots hosts diazotrophs that provide nitrogen in Sorghum bicolor
Source: PLoS Biol. 2025 Mar 3;23(3):e3003037. doi: 10.1371/journal.pbio.3003037 (PMC12136154; doi:10.1371/journal.pbio.3003037)
Supplement: S3 Table — (DOCX) [file pbio.3003037.s009.docx]

**S3 Table.** Mucilage samples from maize and sorghum for primary metabolism analysis

| **Genotype** | **Plant** | **Volume (mg)** |
| --- | --- | --- |
| IS 29091 biological replicate 1 | Sorghum | 1500 |
| IS 29091 biological replicate 2 | Sorghum | 200 |
| IS 29091 biological replicate 3 | Sorghum | 1000 |
| IS 2245 biological replicate 1 | Sorghum | 150 |
| IS 2245 biological replicate 2 | Sorghum | 75 |
| IS 2245 biological replicate 3 | Sorghum | 250 |
| CIMMYT-BANK-017456 biological replicate 1 | Maize | 750 |
| CIMMYT-BANK-017456 biological replicate 2 | Maize | 750 |
| CIMMYT BANK 017456 biological replicate 3 | Maize | 500 |
| CIMMYT BANK 017456 biological replicate 4 | Maize | 500 |
| GRIN AMES 19897 biological replicate 1 | Maize | 400 |
| GRIN AMES 19897 biological replicate 2 | Maize | 500 |
| GRIN AMES 19897 biological replicate 3 | Maize | 250 |
| GRIN AMES 19897 biological replicate 4 | Maize | 100 |
