# Supplementary material for: Mucilage produced by aerial roots hosts diazotrophs that provide nitrogen in Sorghum bicolor
Source: PLoS Biol. 2025 Mar 3;23(3):e3003037. doi: 10.1371/journal.pbio.3003037 (PMC12136154; doi:10.1371/journal.pbio.3003037)
Supplement: S4 Table — Peak (mz/rt) is the mass by charge (mz) divided by the retention time (rt). d.value denotes the comparison between sorghum and maize, where a positive value signifies that the metabolite is more abundant in sorghum than maize and negative values indicate higher abundance in maize. Other acronyms include standard deviation (stdev), raw p-value (rawp), and q-value (q.value) for statistical analysis. (DOCX) [file pbio.3003037.s010.docx]

**S4 Table.** Top metabolites. Peak (mz/rt) is the mass by its charge (mz) divided by the retention time (rt). d.value denotes the comparison between sorghum and maize, where a positive value signifies that the metabolite is more abundant in sorghum compared to maize, and negative values indicate higher abundance in maize. Other acronyms include standard deviation (stdev), raw p-value (rawp) and q-value (q.value) for the statistical analysis.

|  | **Peak (mz/rt)** | **d.value** | **stdev** | **rawp** | **q.value** |
| --- | --- | --- | --- | --- | --- |
| 1 | 4850 | 8.6391 | 22.282 | 0 | 0 |
| 2 | montanic acid | 5.9485 | 1276.4 | 0.00011905 | 0.0032409 |
| 3 | 1064 | 5.825 | 2328.1 | 0.00011905 | 0.0032409 |
| 4 | 4901 | -5.7521 | 469.11 | 0.00015873 | 0.0032409 |
| 5 | 168 | 5.3462 | 3634.3 | 0.00015873 | 0.0032409 |
| 6 | Pinitol | -5.1539 | 375.29 | 0.0002381 | 0.0040512 |
| 7 | Octadecanol | 4.7778 | 248.11 | 0.00043651 | 0.0049514 |
| 8 | *N*-acetyl-aspartate dyethilester | 4.7568 | 18986 | 0.00043651 | 0.0049514 |
| 9 | 84161 | 4.7356 | 631.58 | 0.00043651 | 0.0049514 |
| 10 | 505155 | 4.5206 | 19490 | 0.00059524 | 0.0057391 |
| 11 | 328824 | -4.4684 | 166.07 | 0.00063492 | 0.0057391 |
| 12 | 505146 | 4.4321 | 19227 | 0.0006746 | 0.0057391 |
| 13 | 100956 | -4.2263 | 150.2 | 0.00087302 | 0.0068558 |
| 14 | levoglucosan | 4.0558 | 195.21 | 0.0011111 | 0.0081023 |
| 15 | dodecanol | 3.7188 | 293.75 | 0.001746 | 0.011883 |
| 16 | 1725 | 3.676 | 14665 | 0.0019841 | 0.01263 |
| 17 | 14724 | 3.6622 | 143.57 | 0.0021032 | 0.01263 |
| 18 | azelaic acid | 3.5732 | 108.45 | 0.0025397 | 0.014404 |
| 19 | 103182 | 3.5634 | 85.184 | 0.0026984 | 0.014499 |
| 20 | 21885 | 3.4903 | 1123.9 | 0.0032937 | 0.015836 |
| 21 | thymine | -3.4522 | 216.9 | 0.0034127 | 0.015836 |
| 22 | lignoceric acid | 3.4511 | 465.46 | 0.0034127 | 0.015836 |
| 23 | 127661 | 3.4037 | 393.86 | 0.0037698 | 0.016733 |
| 24 | 2551 | 3.3414 | 130.98 | 0.0043254 | 0.018399 |
| 25 | putrescine | -3.2171 | 1115.5 | 0.0057937 | 0.023659 |
| 26 | threonine | -3.1399 | 2661.8 | 0.0071429 | 0.028046 |
| 27 | 169611 | -3.1161 | 750.57 | 0.0074603 | 0.028208 |
| 28 | 42357 | 3.0631 | 321.56 | 0.0080159 | 0.028218 |
| 29 | 87877 | 3.0619 | 710.68 | 0.0080159 | 0.028218 |
| 30 | 4-hydro-xymandelic acid | 3.0201 | 290.67 | 0.0084921 | 0.028898 |
| 31 | 1-octacosanol | 2.8541 | 963.42 | 0.012143 | 0.035571 |
| 32 | isopalmitic acid | 2.8421 | 218.03 | 0.01246 | 0.035571 |
| 33 | 4-hydroxybenzoic acid | 2.8215 | 5841.1 | 0.012937 | 0.035571 |
| 34 | adipic acid | 2.8159 | 1130.8 | 0.013175 | 0.035571 |
| 35 | 47197 | 2.7985 | 335 | 0.013611 | 0.035571 |
| 36 | 131202 | 2.7821 | 258.41 | 0.014048 | 0.035571 |
