# Supplementary material for: Mucilage produced by aerial roots hosts diazotrophs that provide nitrogen in Sorghum bicolor
Source: PLoS Biol. 2025 Mar 3;23(3):e3003037. doi: 10.1371/journal.pbio.3003037 (PMC12136154; doi:10.1371/journal.pbio.3003037)
Supplement: S5 Table — Number of replicates is indicated in the sample ID. (DOCX) [file pbio.3003037.s011.docx]

**S5 Table.** Maize and sorghum accession for microbiome study. Number of replicates is indicated in the sample ID. UF, University of Florida. WMARS, West Madison Agricultural Research Station.

| **Sample ID** | **Genotype** | **Plant** | **Location** |
| --- | --- | --- | --- |
| S1 (4 samples) | IS 2245 | sorghum | WMARS |
| S2 (3 samples) | IS 2245* | sorghum | WMARS |
| S3 (4 samples) | IS 23992 | sorghum | WMARS |
| S4 (4 samples) | IS 29092 | sorghum | WMARS |
| S5 (4 samples) | IS 2902 | sorghum | UF |
| S6 (4 samples) | IS 2902* | sorghum | UF |
| S7 (4 samples) | IS 2245 | sorghum | UF |
| M1 (4 samples) | CIMMYT-BANK-017456 | maize | WMARS |
| M2 (4 samples) | CIMMYT-BANK-017456 | maize | WMARS |
| M3 (4 samples) | CIMMYTA-BANK-014019 | maize | WMARS |
| M4 (4 samples) | GRIN AMES 19897 | maize | WMARS |
| M5 (3 samples) | GRIN AMES 19897 | maize | WMARS |
| M6 (4 samples) | GRIN AMES 19897 | maize | WMARS |

*Samples were obtained from a low-nitrogen pilot study
