# Supplementary material for: Mucilage produced by aerial roots hosts diazotrophs that provide nitrogen in Sorghum bicolor
Source: PLoS Biol. 2025 Mar 3;23(3):e3003037. doi: 10.1371/journal.pbio.3003037 (PMC12136154; doi:10.1371/journal.pbio.3003037)
Supplement: S6 Table — S, sorghum sample, M, maize sample, and df, degrees of freedom. Statistically significant differences are indicated with asterisks: *p ≤ 0.05; **p ≤ 0.01, and ***p ≤ 0.001. (DOCX) [file pbio.3003037.s012.docx]

**S6 Table.** AMOVA test for each sample comparison. S = sorghum sample, M = maize sample, and df = degrees of freedom. Significant values * ≤ 0.05, ** ≤ 0.01, ***≤ 0.001

| **Group** | **Sum of Square** | **df** | **Mean Square** | **Fixation Index** | **p-value** |
| --- | --- | --- | --- | --- | --- |
| S1-M3 | 0.144504 | 1 | 0.144504 | 5.1731 | 0.005** |
| M4-M5 | 0.0855831 | 1 | 0.0855831 | 2.22751 | 0.095 |
| S2-M4 | 0.0864243 | 1 | 0.0864243 | 3.0695 | 0.038* |
| M2-M5 | 0.115087 | 1 | 0.115087 | 3.71158 | 0.029* |
| S6-S7 | 0.0614475 | 1 | 0.0614475 | 0.486006 | 0.61 |
| S3-M5 | 0.180132 | 1 | 0.180132 | 5.09055 | 0.026* |
| S1-S6 | 0.184116 | 1 | 0.184116 | 1.95325 | 0.263 |
| S5-M6 | 0.450519 | 1 | 0.450519 | 5.24901 | 0.01** |
| S3-M1 | 0.054311 | 1 | 0.054311 | 1.92444 | 0.114 |
| S2-M3 | 0.186426 | 1 | 0.186426 | 7.09958 | 0.03* |
| S2-S7 | 0.266473 | 1 | 0.266473 | 4.39073 | 0.068 |
| S4-S6 | 0.22921 | 1 | 0.22921 | 2.53538 | 0.198 |
| M4-M6 | 0.138883 | 1 | 0.138883 | 3.15016 | 0.05* |
| S2-S6 | 0.137483 | 1 | 0.137483 | 1.29883 | 0.413 |
| S5-M2 | 0.223533 | 1 | 0.223533 | 3.187 | 0.107 |
| M2-M4 | 0.0199211 | 1 | 0.0199211 | 0.70151 | 0.589 |
| M3-M5 | 0.0460803 | 1 | 0.0460803 | 1.26165 | 0.341 |
| M1-M5 | 0.1314 | 1 | 0.1314 | 3.8839 | 0.013* |
| S7-M6 | 0.740479 | 1 | 0.740479 | 10.4001 | 0.02* |
| S6-M2 | 0.225729 | 1 | 0.225729 | 2.42344 | 0.184 |
| S4-M6 | 0.183315 | 1 | 0.183315 | 5.21219 | 0.011* |
| S4-M1 | 0.0626517 | 1 | 0.0626517 | 2.86947 | 0.023* |
| S3-S4 | 0.0656918 | 1 | 0.0656918 | 2.8403 | 0.072 |
| S2-S4 | 0.0574829 | 1 | 0.0574829 | 3.29319 | 0.066 |
| S4-S7 | 0.436925 | 1 | 0.436925 | 8.27984 | 0.029 |
| S5-M3 | 0.407831 | 1 | 0.407831 | 5.45696 | 0.017 |
| S5-M1 | 0.270468 | 1 | 0.270468 | 3.73097 | 0.03* |
| M1-M2 | 0.0309953 | 1 | 0.0309953 | 1.26133 | 0.189 |
| S4-S5 | 0.231663 | 1 | 0.231663 | 3.43716 | 0.019** |
| S3-M3 | 0.16078 | 1 | 0.16078 | 5.27755 | 0.005** |
| S5-S7 | 0.0721351 | 1 | 0.0721351 | 0.697439 | 0.438 |
| S4-M2 | 0.0571673 | 1 | 0.0571673 | 2.93463 | 0.028* |
| M1-M3 | 0.115701 | 1 | 0.115701 | 3.96639 | 0.003** |
| S1-S5 | 0.163211 | 1 | 0.163211 | 2.29049 | 0.184 |
| S4-M5 | 0.0948554 | 1 | 0.0948554 | 3.42189 | 0.034* |
| S7-M5 | 0.549234 | 1 | 0.549234 | 7.74059 | 0.04* |
| S2-S5 | 0.111242 | 1 | 0.111242 | 1.4217 | 0.269 |
| S7-M4 | 0.436884 | 1 | 0.436884 | 7.08226 | 0.001*** |
| S1-M4 | 0.0736667 | 1 | 0.0736667 | 2.49594 | 0.089 |
| S7-M3 | 0.655788 | 1 | 0.655788 | 10.9105 | 0.007** |
| S3-M2 | 0.0690644 | 1 | 0.0690644 | 2.66988 | 0.034* |
| M3-M6 | 0.0679816 | 1 | 0.0679816 | 1.59931 | 0.201 |
| S3-S7 | 0.418749 | 1 | 0.418749 | 7.07855 | 0.011* |
| M5-M6 | 0.10333 | 1 | 0.10333 | 2.07341 | 0.044* |
| S2-M1 | 0.0962802 | 1 | 0.0962802 | 4.08539 | 0.032* |
| M2-M6 | 0.201935 | 1 | 0.201935 | 5.3267 | 0.014* |
| S5-M4 | 0.245334 | 1 | 0.245334 | 3.21468 | 0.023* |
| S5-M5 | 0.348851 | 1 | 0.348851 | 3.94134 | 0.027* |
| S4-M4 | 0.0506137 | 1 | 0.0506137 | 1.97263 | 0.121 |
| S6-M6 | 0.437969 | 1 | 0.437969 | 4.02419 | <0.001* |
| S4-M3 | 0.108391 | 1 | 0.108391 | 4.50182 | 0.012* |
| S2-M2 | 0.0652358 | 1 | 0.0652358 | 3.14503 | 0.025* |
| S6-M1 | 0.272552 | 1 | 0.272552 | 2.85402 | 0.048* |
| S1-S3 | 0.0566845 | 1 | 0.0566845 | 2.10059 | 0.052* |
| M1-M6 | 0.221378 | 1 | 0.221378 | 5.49822 | 0.015* |
| S1-S4 | 0.0218245 | 1 | 0.0218245 | 1.05958 | 0.349 |
| M2-M3 | 0.0991976 | 1 | 0.0991976 | 3.69911 | 0.033* |
| S2-M5 | 0.16442 | 1 | 0.16442 | 5.24333 | 0.105 |
| S1-M5 | 0.123894 | 1 | 0.123894 | 3.83004 | 0.016* |
| S6-M3 | 0.381372 | 1 | 0.381372 | 3.90187 | 0.007** |
| S1-M6 | 0.217747 | 1 | 0.217747 | 5.57941 | 0.009** |
| S2-M6 | 0.264406 | 1 | 0.264406 | 6.68187 | 0.015* |
| S3-S6 | 0.219448 | 1 | 0.219448 | 2.26721 | 0.309 |
| S1-M2 | 0.0740045 | 1 | 0.0740045 | 3.17115 | 0.014* |
| S6-M4 | 0.23653 | 1 | 0.23653 | 2.38145 | 0.211 |
| S7-M1 | 0.485294 | 1 | 0.485294 | 8.38696 | 0.007** |
| S1-S2 | 0.0290423 | 1 | 0.0290423 | 1.31515 | 0.245 |
| M1-M4 | 0.0441517 | 1 | 0.0441517 | 1.43577 | 0.138 |
| M3-M4 | 0.0719655 | 1 | 0.0719655 | 2.18114 | 0.125 |
| S1-S7 | 0.390742 | 1 | 0.390742 | 6.90035 | 0.006** |
| S5-S6 | 0.0159133 | 1 | 0.0159133 | 0.11281 | 0.878 |
| S6-M5 | 0.325414 | 1 | 0.325414 | 2.80247 | 0.157 |
| S3-S5 | 0.194684 | 1 | 0.194684 | 2.63844 | 0.07 |
| S2-S3 | 0.0518463 | 1 | 0.0518463 | 2.06391 | 0.13 |
| S3-M6 | 0.241142 | 1 | 0.241142 | 5.80253 | 0.023* |
| S1-M1 | 0.0850429 | 1 | 0.0850429 | 3.31029 | 0.016* |
| S7-M2 | 0.406276 | 1 | 0.406276 | 7.31907 | 0.032* |
| S3-M4 | 0.0837043 | 1 | 0.0837043 | 2.61203 | 0.001*** |
| All samples S1-S2-S3-S4-S5-S6-S7-M1-M2-M3-M4-M5-M6 | 2.31435 | 12 | 0.192863 | 3.6472 | <0.001*** |
