# Supplementary material for: Mucilage produced by aerial roots hosts diazotrophs that provide nitrogen in Sorghum bicolor
Source: PLoS Biol. 2025 Mar 3;23(3):e3003037. doi: 10.1371/journal.pbio.3003037 (PMC12136154; doi:10.1371/journal.pbio.3003037)
Supplement: S7 Table — (DOCX) [file pbio.3003037.s013.docx]

**S7 Table.** Diazotroph isolated from sorghum mucilage

| **Strain** | **Sorghum Genotype** | **Phylum/Class** | **Closest type strain** | **Length (bp)** | **Similarity (%)** | **Accession Number** |
| --- | --- | --- | --- | --- | --- | --- |
| 1.1 | IS_29091 | *Pseudomonadota/Gammaproteobacteria* | *Stenotrophomonas lactitubi* M15^T^ | 1495 | 99.78 | NR_179509.1 |
| 1.2 | IS_29091 | *Pseudomonadota/Gammaproteobacteria* | *Stenotrophomonas lactitubi* M15^T^ | 1320 | 100 | NR_179509.1 |
| 1.3 | IS_29091 | *Pseudomonadota/Gammaproteobacteria* | *Stenotrophomonas lactitubi* M15^T^ | 1333 | 100 | NR_179509.1 |
| 1.6 | IS_29091 | *Pseudomonadota/Gammaproteobacteria* | *Klebisiella michiganensis* W14^T^ | 1319 | 99.7 | MT572941.1 |
| 1.7 | IS_29091 | *Pseudomonadota/Gammaproteobacteria* | *Stenotrophomonas lactitubi* M15^T^ | 1377 | 99.49 | NR_179509.1 |
| 1.9 | IS_29091 | *Pseudomonadota/Gammaproteobacteria* | *Klebisiella oxitoca* JCM 1665^T^ | 1286 | 99.61 | LC133345.1 |
| 2.1 | IS_31706 | *Pseudomonadota/Betaproteobacteria* | *Pseudacidovorax intermedius* CC-21 | 1298 | 100 | NR_044241.1 |
| 2.2 | IS_31706 | *Actinomycetota/Actinomycetes* | *Microbacterium testaceum* DSM 20166^T^ | 1310 | 100 | NR_026163.1 |
| 2.5 | IS_31706 | *Actinomycetota/Actinomycetes* | *Microbacterium testaceum* DSM 20166^T^ | 1355 | 99.78 | NR_026163.1 |
| 2.6 | IS_31706 | *Pseudomonadota/Gammaproteobacteria* | *Stenotrophomonas rhizophila* e-p10^T^ | 1356 | 99.93 | NR_028930.1 |
| 3.1 | IS_15744 | *Pseudomonadota/Alphaproteobacteria* | *Novosphingobium resinovorum* NCIMB 8767^T^ | 1236 | 100 | NR_044045.1 |
| 3.2 | IS_15744 | *Pseudomonadota/Gammaproteobacteria* | *Stenotrophomonas lactitubi* M15^T^ | 1353 | 99.78 | NR_179509.1 |
| 3.3 | IS_15744 | *Pseudomonadota/Gammaproteobacteria* | *Stenotrophomonas lactitubi* M15^T^ | 1250 | 100 | NR_179509.1 |
| 3.5 | IS_15744 | *Pseudomonadota/Alphaproteobacteria* | *Novosphingobium resinovorum* NCIMB 8767^T^ | 1220 | 100 | NR_044045.1 |
| 3.6 | IS_15744 | *Actinomycetota/Actinomycetes* | *Microbacterium neimengense* 7087^T^ | 1330 | 99.55 | NR_118272.1 |
| 4.2 | IS_31706 | *Actinomycetota/Actinomycetes* | *Microbacterium testaceum* DSM 20166^T^ | 1388 | 99.77 | NR_026163.1 |
| 4.4 | IS_31706 | *Pseudomonadota/Gammaproteobacteria* | *Phytobacter diazotrophicus* DSM 17806^T^ | 1441 | 99.74 | KY288669.1 |
| 4.6 | IS_31706 | *Pseudomonadota/Alphaproteobacteria* | *Sphingobium limneticum* 301^T^ | 1369 | 99.7 | NR_109484.1 |
| 5.1 | IS_31706 | *Pseudomonadota/Alphaproteobacteria* | *Agrobacterium fabacearum* CNPSo 675^T^ | 1295 | 100 | MN741112.1 |
| 5.2 | IS_31706 | *Pseudomonadota/Gammaproteobacteria* | *Pseudoxanthomonas winnipegensis* NML 130738^T^ | 1352 | 99.93 | MH795530.1 |
| 5.3 | IS_31706 | *Bacteroidota/Flavobacteriia* | *Epilithonimonas hungarica* CHB-20p^T^ | 1265 | 99.84 | NR_044354.1 |
| 5.4 | IS_31706 | *Pseudomonadota/Alphaproteobacteria* | *Sphingobium yanoikuyae* HAMBI 1842^T^ | 1317 | 96.82 | LT899948.1 |
| 6.1 | IS_15744 | *Pseudomonadota/Alphaproteobacteria* | *Agrobacterium fabacearum* CNPSo 675^T^ | 1290 | 100 | MN741112.1 |
| 6.11 | IS_15744 | *Pseudomonadota/Gammaproteobacteria* | *Stenotrophomonas nematodicola* W5^T^ | 1365 | 99.93 | NR_181111.1 |
| 6.2 | IS_15744 | *Pseudomonadota/Gammaproteobacteria* | *Stenotrophomonas cyclobalanopsidis* TPQG1-4^T^ | 1356 | 99.85 | MN036524.2 |
| 6.3 | IS_15744 | *Pseudomonadota/Alphaproteobacteria* | *Novosphingobium kaempferiae* Sx8-5^T^ | 1298 | 99.54 | CP089301.1 |
| 6.4 | IS_15744 | *Pseudomonadota/Gammaproteobacteria* | *Stenotrophomonas cyclobalanopsidis* TPQG1-4^T^ | 1356 | 99.7 | MN036524.2 |
| 6.7 | IS_15744 | *Pseudomonadota/Alphaproteobacteria* | *Agrobacterium divergens* LMG 31531^T^ | 1350 | 100 | AM403584.1 |
| 6.8 | IS_15744 | *Pseudomonadota/Alphaproteobacteria* | *Agrobacterium fabacearum* CNPSo 675^T^ | 1292 | 100 | MN741112.1 |
| 6.9 | IS_15744 | *Pseudomonadota/Gammaproteobacteria* | *Stenotrophomonas nematodicola* W5^T^ | 1377 | 99.85 | NR_181111.1 |
| 7.2 | IS_31706 | *Actinomycetota/Actinomycetes* | *Microbacterium oleivorans* BAS69^T^ | 1379 | 98.7 | NR_042262.1 |
| 7.3 | IS_31706 | *Pseudomonadota/Gammaproteobacteria* | *Pseudomonas campi* S1-A32-2 | 1284 | 98.68 | MT415401.1 |
| 7.6 | IS_31706 | *Bacillota/Bacilli* | *Bacillus amyloliquefaciens* DSM 7^T^ | 1389 | 100 | FN597644.1 |
| 7.8 | IS_31706 | *Actinomycetota/Actinomycetes* | *Microbacterium oleivorans* BAS69^T^ | 1329 | 98.87 | NR_042262.1 |
| 8.1 | IS_29314 | *Pseudomonadota/Alphaproteobacteria* | *Azospirillum humicireducens* SgZ-5^T^ | 1181 | 96.21 | CP015285.1 |
| 8.4 | IS_29314 | *Pseudomonadota/Gammaproteobacteria* | *Pseudomonas bharatica* CSV86^T^ | 1331 | 99.77 | MN866057.2 |
| 10.2 | IS_29091 | *Actinomycetota/Actinomycetes* | *Microbacterium neimengense* 7087^T^ | 1291 | 99.84 | NR_118272.1 |
| 10.3 | IS_29091 | *Bacteroidota/Chitinophagia* | *Filimonas endophytica* SR 2-06^T^ | 1292 | 98.14 | NR_145918.1 |
| 10.4 | IS_29091 | *Bacteroidota/Chitinophagia* | *Filimonas zeae* 772^T^ | 1329 | 97.9 | NR_149799.1 |
| 10.5 | IS_29091 | *Actinomycetota/Actinomycetes* | *Microbacterium testaceum* DSM 20166^T^ | 1349 | 100 | NR_026163.1 |
| 10.6 | IS_29091 | *Pseudomonadota/Alphaproteobacteria* | *Agrobacterium divergens* LMG 31531^T^ | 1365 | 100 | AM403584.1 |
| 10.7 | IS_29091 | *Pseudomonadota/Alphaproteobacteria* | *Agrobacterium fabacearum* CNPSo 675^T^ | 1231 | 100 | MN741112.1 |
| 11.1 | IS_15744 | *Actinomycetota/Actinomycetes* | *Microbacterium binotii* CIP 101303^T^ | 1330 | 100 | NR_044290.1 |
| 11.1 | IS_15744 | *Actinomycetota/Actinomycetes* | *Microbacterium testaceum* DSM 20166^T^ | 1338 | 99.18 | NR_026163.1 |
| 11.2 | IS_15744 | *Pseudomonadota/Alphaproteobacteria* | *Sphingobium yanoikuyae* HAMBI 1842^T^ | 1297 | 99.92 | LT899948.1 |
| 11.4 | IS_15744 | *Pseudomonadota/Gammaproteobacteria* | *Pseudomonas sediminis* PI11^T^ | 1145 | 94.53 | KP319033.1 |
| 11.7 | IS_15744 | *Pseudomonadota/Alphaproteobacteria* | *Azospirillum palustre* B2^T^ | 1232 | 99.35 | NR_178321.1 |
| 11.8 | IS_15744 | *Pseudomonadota/Alphaproteobacteria* | *Sphingobium yanoikuyae* HAMBI 1842^T^ | 1311 | 100 | LT899948.1 |
| 12.2 | PI_534063 | *Pseudomonadota/Alphaproteobacteria* | *Rhizobium cellulosilyticum* ALA10B2 | 1183 | 99.32 | NR_043985.1 |
| 12.4 | PI_534063 | *Pseudomonadota/Betaproteobacteria* | *Herbaspirillum seropedicae* Z67^T^ | 1388 | 99.71 | CP011930.1 |
| 12.6 | PI_534063 | *Actinomycetota/Actinomycetes* | *Microbacterium neimengense* 7087^T^ | 1378 | 99.78 | NR_118272.1 |
| 12.7 | PI_534063 | *Pseudomonadota/Betaproteobacteria* | *Herbaspirillum seropedicae* Z67^T^ | 1341 | 99.85 | CP011930.1 |
| 12.9 | PI_534063 | *Pseudomonadota/Betaproteobacteria* | *Herbaspirillum seropedicae* Z67^T^ | 1281 | 99.84 | CP011930.1 |
| 13.1 | IS_9745 | *Actinomycetota/Actinomycetes* | *Microbacterium testaceum* DSM 20166^T^ | 1379 | 99.78 | NR_026163.1 |
| 13.3 | IS_9745 | *Actinomycetota/Actinomycetes* | *Microbacterium hominis* DSM 12509^T^ | 1325 | 98.19 | NR_042480.1 |
| 13.4 | IS_9745 | *Actinomycetota/Actinomycetes* | *Microbacterium hominis* DSM 12509^T^ | 1360 | 99.76 | NR_042480.1 |
| 13.5 | IS_9745 | *Pseudomonadota/Gammaproteobacteria* | *Stenotrophomonas terrae* R-32768 | 1361 | 99.34 | NR_042569.1 |
| 13.7 | IS_9745 | *Actinomycetota/Actinomycetes* | *Microbacterium testaceum* DSM 20166^T^ | 1305 | 99.16 | NR_026163.1 |
| 13.8 | IS_9745 | *Actinomycetota/Actinomycetes* | *Microbacterium testaceum* DSM 20166^T^ | 1322 | 99.17 | NR_026163.1 |
| 13.9 | IS_9745 | *Pseudomonadota/Gammaproteobacteria* | *Stenotrophomonas lactitubi* M15^T^ | 1339 | 99.78 | NR_179509.1 |
| 14.1 | IS_29091 | *Actinomycetota/Actinomycetes* | *Microbacterium testaceum* DSM 20166^T^ | 1270 | 98.98 | NR_026163.1 |
| 14.2 | IS_29091 | *Pseudomonadota/Gammaproteobacteria* | *Pseudomonas turukhanskensis* IB1.1^T^ | 1331 | 98.5 | NR_152710.1 |
| 14.3 | IS_29091 | *Pseudomonadota/Gammaproteobacteria* | *Enterobacter cancerogenus* FDAARGOS 1428^T^ | 1287 | 99.92 | CP077290.1 |
| 14.4 | IS_29091 | *Actinomycetota/Actinomycetes* | *Microbacterium aerolatum* CCM 4955^T^ | 1289 | 99.07 | MT760116.1 |
| 14.5 | IS_29091 | *Actinomycetota/Actinomycetes* | *Microbacterium testaceum* DSM 20166^T^ | 1373 | 99.05 | NR_026163.1 |
| 14.6 | IS_29091 | *Actinomycetota/Actinomycetes* | *Microbacterium testaceum* DSM 20166^T^ | 1355 | 99.12 | NR_026163.1 |
| 14.7 | IS_29091 | *Pseudomonadota/Gammaproteobacteria* | *Pseudomonas turukhanskensis* IB1.1^T^ | 1299 | 100 | NR_152710.1 |
| 14.9 | IS_29091 | *Actinomycetota/Actinomycetes* | *Microbacterium testaceum* DSM 20166^T^ | 1315 | 100 | NR_026163.1 |
| 15.1 | IS_13971 | *Actinomycetota/Actinomycetes* | *Microbacterium laevaniformans* DSM 20140^T^ | 1270 | 99.21 | MN543870.1 |
| 15.2 | IS_13971 | *Pseudomonadota/Alphaproteobacteria* | *Novosphingobium resinovorum* NCIMB 8767^T^ | 1257 | 100 | EF029110.2 |
| 15.5 | IS_13971 | *Pseudomonadota/Alphaproteobacteria* | *Devosia riboflavina* NBRC 13584^T^ | 1191 | 100 | NR_113618.1 |
| 15.7 | IS_13971 | *Pseudomonadota/Alphaproteobacteria* | *Ketogulonicigenium vulgare* DSM 4025T | 1259 | 100 | DQ915606.1 |
| 15.8 | IS_13971 | *Pseudomonadota/Alphaproteobacteria* | *Devosia riboflavina* NBRC 13584^T^ | 1347 | 99.85 | NR_113618.1 |
| 15.9 | IS_13971 | *Pseudomonadota/Betaproteobacteria* | *Pseudacidovorax intermedius* CC-21 | 1213 | 96.83 | NR_044241.1 |
| 16.1 | IS_15744 | *Actinomycetota/Actinomycetes* | *Microbacterium oleivorans* BAS69^T^ | 1269 | 99.13 | NR_042262.1 |
| 16.2 | IS_15744 | *Actinomycetota/Actinomycetes* | *Microbacterium oleivorans* BAS69^T^ | 1276 | 98.82 | NR_042262.1 |
| 16.3 | IS_15744 | *Pseudomonadota/Alphaproteobacteria* | *Sphingobium yanoikuyae* HAMBI 1842^T^ | 1355 | 99.92 | LT899948.1 |
| 16.4 | IS_15744 | *Actinomycetota/Actinomycetes* | *Microbacterium binotii* CIP 101303^T^ | 1319 | 100 | NR_044290.1 |
| 16.5 | IS_15744 | *Actinomycetota/Actinomycetes* | *Microbacterium oleivorans* BAS69^T^ | 1377 | 98.77 | NR_042262.1 |
| 16.7 | IS_15744 | *Actinomycetota/Actinomycetes* | *Microbacterium neimengense* 7087^T^ | 1370 | 99.89 | NR_118272.1 |
| 17.2 | IS_29091 | *Pseudomonadota/Alphaproteobacteria* | *Agrobacterium divergens* LMG 31531^T^ | 1231 | 100 | AM403584.1 |
| 17.3 | IS_29091 | *Pseudomonadota/Alphaproteobacteria* | *Novosphingobium kaempferiae* Sx8-5^T^ | 1193 | 99.92 | CP089301.1 |
| 17.4 | IS_29091 | *Pseudomonadota/Gammaproteobacteria* | *Phytobacter diazotrophicus* DSM 17806^T^ | 1288 | 99.74 | KY288669.1 |
| 17.5 | IS_29091 | *Pseudomonadota/Alphaproteobacteria* | *Agrobacterium divergens* LMG 31531^T^ | 1291 | 98.99 | AM403584.1 |
| 17.6 | IS_29091 | *Pseudomonadota/Gammaproteobacteria* | *Pseudomonas turukhanskensis* IB1.1^T^ | 1331 | 98.81 | NR_152710.1 |
| 18.1 | IS_31706 | *Pseudomonadota/Gammaproteobacteria* | *Enterobacter cancerogenus* FDAARGOS 1428^T^ | 1247 | 99.68 | CP077290.1 |
| 18.2 | IS_31706 | *Pseudomonadota/Alphaproteobacteria* | *Agrobacterium larrymoorei* ATCC 51759^T^ | 1230 | 100 | MT780298.1 |
| 18.3 | IS_31706 | *Pseudomonadota/Alphaproteobacteria* | *Sphingobium yanoikuyae* HAMBI 1842^T^ | 1320 | 100 | LT899948.1 |
| 18.4 | IS_31706 | *Pseudomonadota/Gammaproteobacteria* | *Stenotrophomonas maltophilia* IAM 12423 | 1295 | 99.85 | MN240936.1 |
| 18.6 | IS_31706 | *Pseudomonadota/Alphaproteobacteria* | *Novosphingobium kaempferiae* Sx8-5^T^ | 1311 | 99.39 | CP089301.1 |
| 18.7 | IS_31706 | *Pseudomonadota/Gammaproteobacteria* | *Stenotrophomonas lactitubi* M15^T^ | 1404 | 99.52 | NR_179509.1 |
| 18.8 | IS_31706 | *Pseudomonadota/Alphaproteobacteria* | *Azospirillum palustre* B2^T^ | 1267 | 98.97 | NR_178321.1 |
| 18.9 | IS_31706 | *Pseudomonadota/Alphaproteobacteria* | *Azospirillum humicireducens* SgZ-5^T^ | 1298 | 99.03 | CP015285.1 |
| 19.2 | IS_31706 | *Bacteroidota/Flavobacteriia* | *Epilithonimonas hungarica* CHB-20p^T^ | 1339 | 99.71 | NR_044354.1 |
| 19.4 | IS_31706 | *Pseudomonadota/Gammaproteobacteria* | *Stenotrophomonas cyclobalanopsidis* TPQG1-4^T^ | 1403 | 99.86 | MN036524.2 |
| 19.6 | IS_31706 | *Bacteroidota/Chitinophagia* | *Siphonobacter intestinalis* 63MJ-2 | 1335 | 99.1 | NR_165683.1 |
| 19.7 | IS_31706 | *Pseudomonadota/Gammaproteobacteria* | *Klebsiella variicola* DSM 15968 | 1410 | 99.65 | CP010523.2 |
| 20.1 | IS_15744 | *Pseudomonadota/Alphaproteobacteria* | *Sphingobium yanoikuyae* HAMBI 1842^T^ | 1311 | 100 | LT899948.1 |
| 20.2 | IS_15744 | *Pseudomonadota/Alphaproteobacteria* | *Sphingobium yanoikuyae* HAMBI 1842^T^ | 1299 | 100 | LT899948.1 |
| 20.3 | IS_15744 | *Actinomycetota/Actinomycetes* | *Microbacterium testaceum* DSM 20166^T^ | 1373 | 99.16 | NR_026163.1 |
| 20.4 | IS_15744 | *Pseudomonadota/Alphaproteobacteria* | *Agrobacterium pusense* NRCPB10^T^ | 1291 | 99.92 | NR_116874.1 |
| 21.1 | IS_29091 | *Pseudomonadota/Alphaproteobacteria* | *Shinella oryzae* Z-25^T^ | 1341 | 98.44 | NR_029103.1 |
| 21.3 | IS_29091 | *Pseudomonadota/Gammaproteobacteria* | *Stenotrophomonas lactitubi* M15^T^ | 1293 | 99.61 | NR_179509.1 |
