# Supplementary material for: Mucilage produced by aerial roots hosts diazotrophs that provide nitrogen in Sorghum bicolor
Source: PLoS Biol. 2025 Mar 3;23(3):e3003037. doi: 10.1371/journal.pbio.3003037 (PMC12136154; doi:10.1371/journal.pbio.3003037)
Supplement: S8 Table — References to the origin of these strains and the source of isolation are provided. (DOCX) [file pbio.3003037.s014.docx]

**S8 Table.** Diazotrophs used for biological nitrogen fixation experiments. References to the origin of these strains and the source of isolation are provided

| **Diazotroph** | **Phenotype** | **Reference** | **Source*** |
| --- | --- | --- | --- |
| *Azospirillum brasilense* FP2 ^#^ | Fixer strain | [[1]](https://paperpile.com/c/zEOMyT/zQZS) | Wheat, maize, and forage grass (*Poa pratense*) |
| *Azospirillum baldaniorum* Sp 245^T#^ | Fixer strain | [[2]](https://paperpile.com/c/zEOMyT/m2W4) | Wheat |
| *Klebisiela variicola* A3^#^ | Fixer strain | This study | Sorghum mucilage |
| *Klebisiela michiganensis* A2 ^#^ | Fixer strain | This study | Maize mucilage |
| *Azorhizobium caulinodans* ORS571^T #^ | Fixer strain | [[3]](https://paperpile.com/c/zEOMyT/CAGE) | Nodules in *Sesbania rostrata* |
| *Stutzerimonas stutzeri* A1501 ^#^ | Fixer strain | [[4]](https://paperpile.com/c/zEOMyT/rwlJ) | Rice |
| *Paraburkholderia silvatlantica* SRMrh-20^T #^ | Fixer strain | [[5]](https://paperpile.com/c/zEOMyT/paRM) | Maize |
| *Azotobacter vinelandii* DJ^#^ | Fixer strain | [[6]](https://paperpile.com/c/zEOMyT/eiCW) | Ubiquitous |
| *Herbaspirillum seropedicae* SmR1^#^ | Fixer strain | [[5]](https://paperpile.com/c/zEOMyT/paRM) | Maize, sorghum, rice, wheat and sugarcane |
| *Azospirillum brasilense* FP10^#^ | Non-fixer strain | [[1]](https://paperpile.com/c/zEOMyT/zQZS) | Wheat, maize, and forage grass (*Poa pratense*) |
| *Azotobacter vinelandii* DJ100 Δ*nifD* | Non-fixer strain | [[7]](https://paperpile.com/c/zEOMyT/GUvy) | Ubiquitous |
| *Azorhizobium caulinodans* ORS571 Δ*nifA* | Non-fixer strain | [[8]](https://paperpile.com/c/zEOMyT/d9Qs) | Nodules in *Sesbania rostrata* |

* Plant(s) where it has been isolated.

^#^ For the ARA experiment

**Reference:**

1. [Pedrosa FO, Yates MG. Regulation of nitrogen fixation (*nif*) genes of *Azospirillum brasilense* by nifA and *ntr* (*gln)* type gene products. FEMS Microbiol Lett. 1984;23: 95–101.](http://paperpile.com/b/zEOMyT/zQZS)

2. [Baldani VLD, Baldani JI, Döbereiner J. Effects of *Azospirillum* inoculation on root infection and nitrogen incorporation in wheat. Can J Microbiol. 1983;29: 924–929.](http://paperpile.com/b/zEOMyT/m2W4)

3. [Dreyfus B, Dommergues Y. Nitrogen-fixing nodules induced by *Rhizobium* on the stem of the tropical legume *Sesbania rostrata*. FEMS Microbiol Lett. 1981;10: 313–317.](http://paperpile.com/b/zEOMyT/CAGE)

4. [Qiu Y, Zhou S, Mo X, You C, Wang D. Investigation of dinitrogen fixation bacteria isolated from rice rhizosphere. Chinese Sc bull. 1981;26: 383–384.](http://paperpile.com/b/zEOMyT/rwlJ)

5. [Perin L, Martínez-Aguilar L, Paredes-Valdez G, Baldani JI, Estrada-de Los Santos P, Reis VM, et al. *Burkholderia silvatlantica* sp. nov., a diazotrophic bacterium associated with sugar cane and maize. Int J Syst Evol Microbiol. 2006;56: 1931–1937.](http://paperpile.com/b/zEOMyT/paRM)

6. [Setubal JC, dos Santos P, Goldman BS, Ertesvåg H, Espin G, Rubio LM, et al. Genome sequence of *Azotobacter vinelandii*, an obligate aerobe specialized to support diverse anaerobic metabolic processes. J Bacteriol. 2009;191: 4534–4545.](http://paperpile.com/b/zEOMyT/eiCW)

7. [Robinson AC, Burgess BK, Dean DR. Activity, reconstitution, and accumulation of nitrogenase components in *Azotobacter vinelandii* mutant strains containing defined deletions within the nitrogenase structural gene cluster. J Bacteriol. 1986;166: 180–186.](http://paperpile.com/b/zEOMyT/GUvy)

8. [Ryu M-H, Zhang J, Toth T, Khokhani D, Geddes BA, Mus F, et al. Control of nitrogen fixation in bacteria that associate with cereals. Nat Microbiol. 2020;5: 314–330.](http://paperpile.com/b/zEOMyT/d9Qs)
